# A Combined Model to Improve the Prediction of Local Control for Lung Cancer Patients Undergoing Stereotactic Body Radiotherapy Based on Radiomic Signature Plus Clinical and Dosimetric Parameters

**DOI:** 10.3389/fonc.2021.819047

**Published:** 2022-01-31

**Authors:** Li-Mei Luo, Bao-Tian Huang, Chuang-Zhen Chen, Ying Wang, Chuang-Huang Su, Guo-Bo Peng, Cheng-Bing Zeng, Yan-Xuan Wu, Ruo-Heng Wang, Kang Huang, Zi-Han Qiu

**Affiliations:** ^1^ Department of Radiation Oncology, Shantou University Medical College, Shantou, China; ^2^ Department of Radiation Oncology, Cancer Hospital of Shantou University Medical College, Shantou, China; ^3^ Department of Radiation Oncology, Shantou Central Hospital, Shantou, China; ^4^ Department of Radiation Oncology, Meizhou People’s Hospital (Huangtang Hospital), Meizhou Academy of Medical Sciences, Meizhou, China; ^5^ Department of Otolaryngology-Head and Neck Surgery, The First Affiliated Hospital of Shantou University Medical College, Shantou, China

**Keywords:** lung cancer, stereotactic body radiotherapy, local control, radiomics, clinical, dosimetric, prediction model

## Abstract

**Purpose:**

Stereotactic body radiotherapy (SBRT) is an important treatment modality for lung cancer patients, however, tumor local recurrence rate remains some challenge and there is no reliable prediction tool. This study aims to develop a prediction model of local control for lung cancer patients undergoing SBRT based on radiomics signature combining with clinical and dosimetric parameters.

**Methods:**

The radiomics model, clinical model and combined model were developed by radiomics features, incorporating clinical and dosimetric parameters and radiomics signatures plus clinical and dosimetric parameters, respectively. Three models were established by logistic regression (LR), decision tree (DT) or support vector machine (SVM). The performance of models was assessed by receiver operating characteristic curve (ROC) and DeLong test. Furthermore, a nomogram was built and was assessed by calibration curve, Hosmer-Lemeshow and decision curve.

**Results:**

The LR method was selected for model establishment. The radiomics model, clinical model and combined model showed favorite performance and calibration (Area under the ROC curve (AUC) 0.811, 0.845 and 0.911 in the training group, 0.702, 0.786 and 0.818 in the validation group, respectively). The performance of combined model was significantly superior than the other two models. In addition, Calibration curve and Hosmer-Lemeshow (training group: P = 0.898, validation group: P = 0.891) showed good calibration of combined nomogram and decision curve proved its clinical utility.

**Conclusions:**

The combined model based on radiomics features plus clinical and dosimetric parameters can improve the prediction of 1-year local control for lung cancer patients undergoing SBRT.

## Introduction

Lung cancer is the second most common cancer and the main cause of cancer-related deaths, more than 2.21 million patients worldwide are affected every year (1). With the improvement of radiotherapy technology, stereotactic body radiation therapy (SBRT) is generally recognized as a standard option for early stage lung cancer patients who are not fit or healthy enough to be candidates for surgery or who refuse operation due to various complications (2). SBRT is also well established in the treatment of oligometastatic patients, e.g., with pulmonary metastases (3). This precise modality uses high doses to ablative cancer target with low doses to protect surrounding tissue. The local control rate in 5 years after SBRT is about 72% with a median follow-up of 4 years for early stage localized tumors (4). Furthermore, a variety of studies have reported local control is excellent after SBRT; however, there are still patients suffering from local recurrence (5). Therefore, a model for accurately and individually predicting the local control status for lung cancer patients after SBRT is highly desirable.

The maximum standardized uptake value (SUVmax) in PET-CT was used to predict local recurrence after SBRT, but the results varied from institutions to patient groups, suggesting that its prognostic value was uncertain (6, 7). Several studies reported some clinical and dosimetric factors were influential parameters for local control prediction (8–11) and dose-response model to calculate local control possibility for lung SBRT patients employed clinical and dosimetric parameters were established (12–15). However, their models did not accurately predict patients’ outcome, while other tumor individual characteristics were not considered. A comprehensive and noninvasive approach based on individual heterogeneous to screen candidate patients with tumor local control status is necessary.

Radiomics is based on the extraction of tumor features from traditional medical images to predict treatment effectiveness and prognosis of different diseases, including lung cancer, esophageal cancer, and prostate cancer (16–19). Moreover, radiomics has prognostic value in predicting clinical outcomes of pulmonary SBRT (20, 21). However, few prediction models considering radiomic signature combined with clinical and dosimetric parameters has been proposed to evaluate the tumor local control for lung cancer patients undergoing SBRT.

Therefore, the aim of our study is to generate a robust combined model for predicting 1-year tumor local control in primary and secondary lung cancer patients treated with SBRT by integrating radiomic signature and clinical and dosimetric parameters.

## Materials and Methods

**Figure 1:**
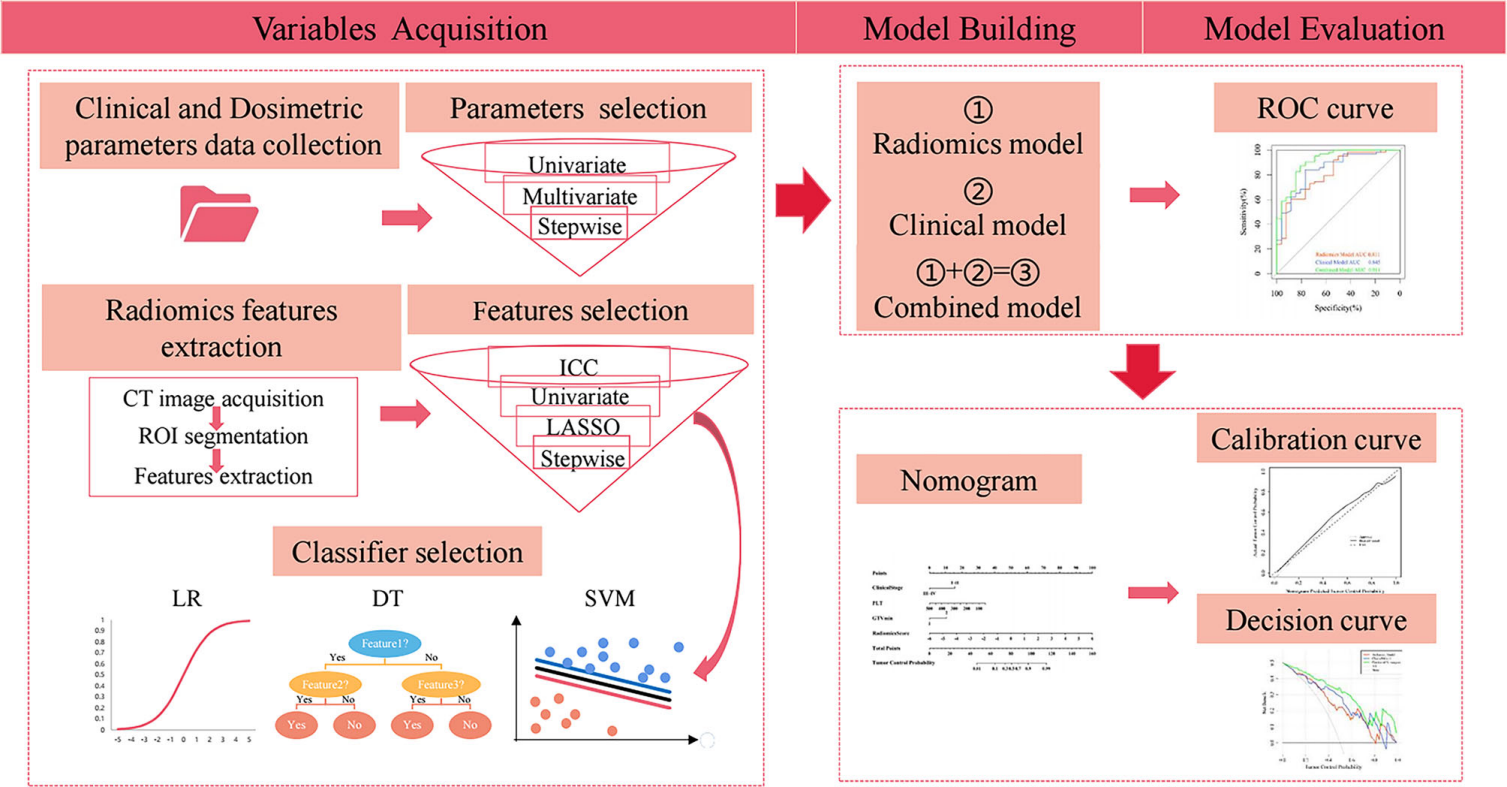
Workflow of the study. ROI, region of interest; ICC, intraclass correlation coefficient; LASSO, least absolute shrinkage and selection operator; LR, logistic regression; DT, decision tree; SVM, support vector machine. ROC, receiver operating characteristic.

The workflow of the study is shown in **Figure 1**.

### Patients’ Population and Treatment

We firstly analyzed retrospectively registered data from July 27, 2011 to December 7, 2018 of patients diagnosed with primary and secondary lung cancer and treated with lung SBRT in Cancer Hospital of Shantou (*N* = 134). Next, some patients with irradiation sites including chest wall, mediastinum, and thoracic vertebra (*N* = 4), who were lost to follow-up (*N* = 18) and did not complete the treatment course (*N* = 1) were excluded from the analysis. Finally, 119 patients with 18 patients had repeat lesions and 129 tumors were available for next analysis. The study was conducted in accordance with the Declaration of Helsinki and approved by the ethical board; however, patient written informed consent was waived. Our patients were staged by using the seventh editions of the AJCC staging system. Tumors were simulated *via* four-dimensional computed tomography (4DCT) or three-dimensional computed tomography (3DCT). The internal target volume (ITV) with 4DCT was defined by combining gross tumor volumes (GTVs) contoured at 10 respiratory phases. In addition, some ITV with 3DCT was defined by two GTVs contoured at the peak-exhale and peak-inhale respiratory phases; other ITV with 3DCT was defined by observing the tumor motion amplitude obtained from fluoroscopy. The planning target volume (PTV) was delineated by adding 5 mm of ITV in all directions. Cone beam computed tomography (CBCT) was used for image guidance and tumor localization before each fraction with correction. All patients were randomly assigned to the training group and validation group in a ratio of 7:3.

### Follow-Up

Patients were evaluated with CT scans repeated every 3 months after the treatment in the first year, and then every 6 months thereafter (4). Tumor local control is defined as the absence of local recurrence of the tumor at the treatment site. Local recurrence was defined by pathologic confirmation, mass progression of the primary tumor, and the involved lobe on two consecutive CT at least 6 months, or the discretion of oncologists based on clinical symptoms and signs of patients (14, 22).

### Clinical and Dosimetric Parameters Data Collection

We collected patients’ baseline clinical and dosimetric parameters and 1-year tumor local status after SBRT. All the doses mentioned in this paper were biologically effective doses (BEDs), the linear-quadratic model with an α/β ratio of 10 Gy was adopted for calculating BEDs, BED = *n* × *d* × [1 + *d*/(*α*/*β*)], where *n* is the fraction number and *d* is the fractional dose (15). Clinical data included gender, age, smoking status, Karnofsky performance status (KPS), body mass index (BMI), clinical stage, location, histology, equivalent diameter, GTV, PTV, chemotherapy or not, lymphocyte, neutrophil, platelet (PLT), neutrophil-to-lymphocyte ratio (NLR), platelet-to-lymphocyte ratio (PLR), hemoglobin (Hb), tumor site, immobilization device, and 4DCT or not. Dosimetric data included the prescription dose that covers 95% of the target area expressed as BED (D_95_) and the maximum dose in the whole plan (D_max_); the minimum dose of PTV (PTV_min_), mean dose of PTV (PTV_mean_), and maximum dose of PTV (PTV_max_) and dose inhomogeneity in PTV (PTV_min_/PTV_max_); and the minimum dose of GTV (GTV_min_), mean dose of GTV (GTV_mean_), and the maximum dose of GTV (GTV_max_) and dose inhomogeneity in GTV (GTV_min_/GTV_max_).

### Clinical and Dosimetric Parameter Selection

The univariate logistic regression (LR) analysis was applied to evaluate whether parameters were candidate predictors of 1-year tumor local control and a stepwise multivariate LR was used to determine the best variables of statistically significant parameters in univariate analysis.

### CT Image Acquisition, Region of Interest Segmentation and Quantitative Radiomics Features Extraction

All patients underwent CT scanning prior to SBRT treatment using a Brilliance Big Bore CT (Philips Brilliance CT Big Bore Oncology Configuration, Cleveland, OH, USA). The detailed information of the CT scanners was as follows: tube voltage of 120 kVp, tube current of 350 mA, convolution kernel of standard, and construction matrix of 512 × 512. The scanning range was from the apex to the bottom of the lung. CT images were then transferred to an Eclipse treatment planning system (Version 10.0, Varian Medical System, Inc., Palo Alto, CA, USA) for the whole tumor delineation, also known as the region of interest (ROI) segmentation by one radiology doctor with more than 10 years of work experience (23).

Radiomics features were automatically extracted from each tumor segmentation using PyRadiomics (https://github.com/Radiomics/pyradiomics). The images which were used to extract the radiomics features could be either the original image or the derivative filtered images including Laplacian of Gaussian (LoG), Wavelet, Square, SquareRoot, Logarithm. Collectively, the feature types extracted from each image type include shape features provided the geometric volume of ROI, first-order features described the individual voxel value distribution in the intensity histogram of ROI, texture features reflected the organization and arrangement of the surface structure with slow change or periodic change, including gray-level co-occurrence matrix (GLCM), gray-level dependence matrix (GLDM), gray-level run length matrix (GLRLM), gray-level size zone matrix (GLSZM), and neighborhood gray-tone difference matrix (NGTDM) (24). In order to ensure the repeatability of the results, the images and features were resampled and z-score normalized respectively.

### Radiomics Feature Selection

A large number of radiomic features may result in overfitting of the model, reducing the predictive performance of the model. To overcome the dimensional disaster and reduce the bias caused by many radiomics features, we gradually use four methods of the intraclass correlation coefficient (ICC), univariant analysis, least absolute shrinkage and selection operator (LASSO), and stepwise regression to select the vital features from the training group. First, in order to minimize the differences between observers and enhance the robustness of features, two radiology doctors independently delineated 30 randomly chosen samples drawn from patients, and then the ICC was calculated from the extracted features of these 30 cases to assess intraobserver and interobserver reproducibility. The features with ICC >0.75 were considered stable for the further analysis. The ICC was conducted by using the “irr” package in R software (25). Subsequently, we evaluated remaining radiomics features using the independent samples *t*-test or the Mann-Whitney *U* test to collect statistically significant features with a *p*-value of <0.05. In addition, to deal with the high-dimensional data and enhance the prediction accuracy, the LASSO regression, as an effective dimensionality reduction method, was applied to select potentially important features by regularizing concurrently. The optimal area under curve and parameter log (*λ*) were determined through 10-fold cross-validation to control the complexity of the model and select the most robust and nonredundant radiomics features (26, 27). The LASSO logistic regression was conducted by using the “glmnet” package (28). Finally, stepwise regression is used to eliminate the redundant features to avoid the multicollinearity. Moreover, the importance of the most valuable features were analyzed and evaluated by correlation analysis.

### Classifier Selection

We fed the final selected radiomics features into the classifiers to build the optimal radiomics model. In our study, LR, decision tree (DT), and support vector machine (SVM) were used to build and evaluate radiomics model, and the best classification method was selected for subsequent analysis (29–31). The DT model was performed using the “rpart” package (32), while the “e1071” package was employed to develop the SVM model (28), all of them are carried out by tuning the parameters. To complement the analyses, the radiomics signature (radiomics score) was calculated using the radiomics features. The best classifier was adopted for building clinical model and combined model.

### Prediction Model Development

Accordingly, three different prediction models were described briefly as: the radiomics model composed of radiomics signature, the clinical model constructed from clinical and dosimetric parameters, and the combined model developed by combining radiomics signature and clinical and dosimetric parameters.

### Model Performance Comparison

Based on receiver operating characteristic (ROC) curve, the prediction models were compared by calculating the area under the ROC curve (AUC) values, *p*-value, accuracy, sensitivity, specificity, and DeLong test. The ROC curves were plotted based on the “pROC” package (33).

### Nomogram Construction and Validation

In order to visually and individually predict the tumor control probability (TCP) of lung cancer after SBRT, we created a nomogram which was developed by the prediction model with the best performance in the training group. The ability of the nomogram was conducted by the calibration curve and the Hosmer-Lemeshow test. The net benefits and the clinical usefulness of three models for prognosis was measured and compared by the decision curve analysis. The nomogram and the DCA were plotted using the “rms” package and the “dca.R.” package, respectively (33).

### Statistical Analysis

Statistical analyses were performed based on SPSS v.23.0 (SPSS Inc., Chicago, IL, USA) and R software v.4.0.2 (R Project for Statistical Computing, Vienna, Austria). The Student’s *t*-test or Mann-Whitney *U* test was employed to compare continuous variables, and the Chi-square test or Fisher’s exact test was applied for categorical variables. The optimal cutoff point was assessed by using the Youden’s index on the ROC curve (34). An AUC comparison of the three prediction models with the best classifier methods was performed by DeLong test. The tests were two-sided, and *p*-values less than 0.05 were considered statistically significant.

## Results

### Clinical and Dosimetric Parameters of Patients

**Figure 2:**
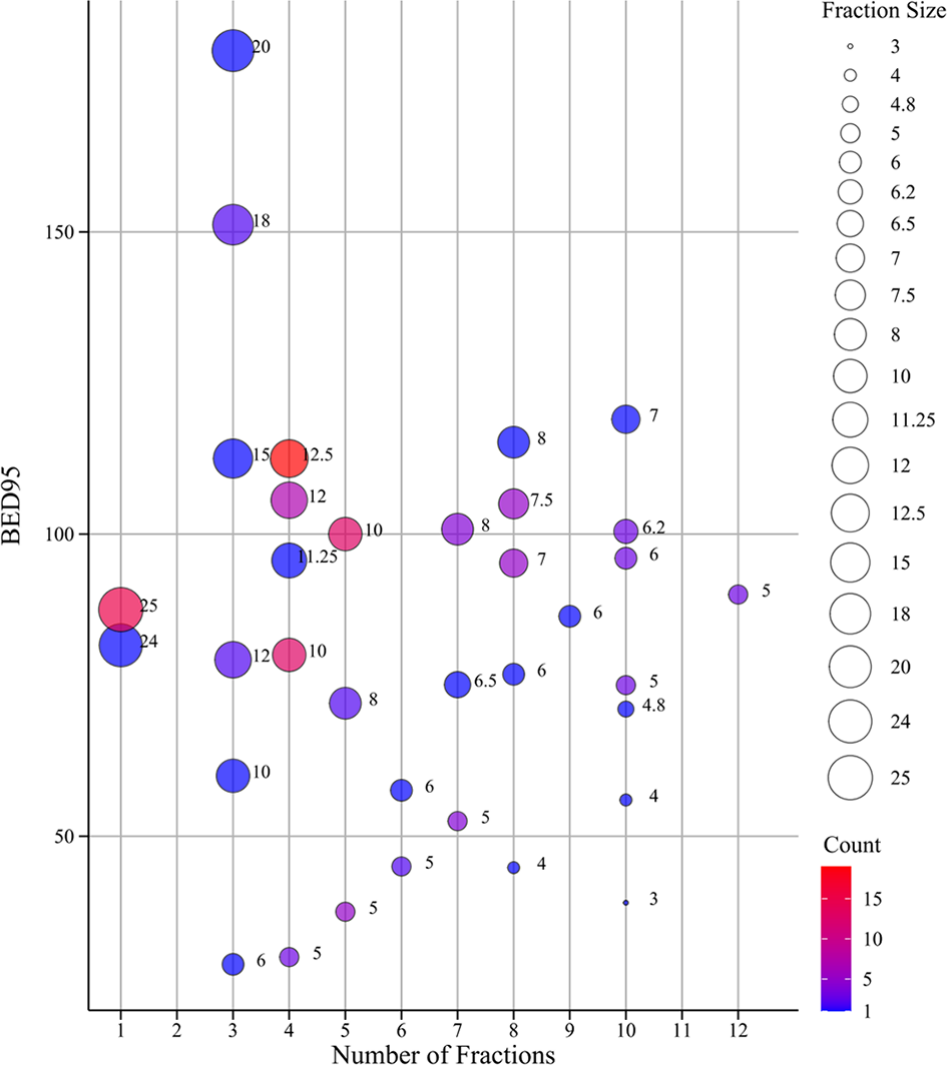
The bubble chart of fractionation schemes and BED_95_. The size of dots indicates the size of fraction; the different color of dots indicates different count ranges. BED_95_, the prescription dose covers 95% of the target area expressed as BED.

Data for 129 tumors from 111 primary and secondary lung cancer patients treated with SBRT were available, of which 89 and 40 tumors were divided into the training and validation group, respectively. Baseline characteristics are presented on **Table S1**. Males constituted 93 (72.1%) of the sample. Mean age was 62 years. Most tumors (82.9%) were at a peripheral location compared with other location. A minority of tumors (18.6%) were treated with combined radiotherapy and concurrent chemotherapy. The median prescription dose was 48 Gy (range 18–70) delivered in a median of 4 fractions (range 1–12), and the median prescription dose in BED_95_ was 95.2 Gy (range 28.8–180). The distribution of fractionation schemes and BED_95_ used in our study was described in **Figure 2**; most used dose-fractionation scheme was 50 Gy in 4 fractions. One year after SBRT treatment, 91 tumors were local controlled and 38 local failures were observed. The optimal cutoff values of dosimetric parameters based on ROC curve are shown in **Table 1**. No significant association was seen with baseline clinical and dosimetric characteristics in the training group and validation group of tumors. The balance of the two sets of data suggests that the patient grouping was reasonable.

**Table 1:** The optimal cut-off values of dosimetric parameters.

Dosimetric parameters	BED_95_	BED_max_	BEDPTV_min_	BEDPTV_max_	BEDPTV_mean_	BEDGTV_min_	BEDGTV_max_	BEDGTV_mean_
Cutoff values	84.00	110.85	80.43	110.85	101.73	98.79	103.87	97.06

BED_95_, the prescription dose covers 95% of the target area expressed as BED; BED_max_, the maximum dose in the whole plan; BEDPTV_min_, the minimum dose of PTV; BEDPTV_mean_, mean dose of PTV; BEDPTV_max_, the maximum dose of PTV; BEDGTV_min_, the minimum dose of GTV; BEDGTV_mean_, mean dose of GTV; BEDGTV_max_, the maximum dose of GTV.

### Clinical and Dosimetric Parameter Selection

The relationship between clinical and dosimetric parameters and the 1-year tumor local status of primary and secondary lung cancer after SBRT for the training group is summarized in **Table 2**. In the univariate analysis, clinical stage status, history, PLT, PLR, Hb, D_95_, D_max_, PTV_min_, PTV_max_, PTV_mean_, GTV_min_, GTV_max_, GTV_mean_, and PTV_min_/PTV_max_ were found to be significantly different between the 1-year local control status and the local failure status (all *p* < 0.05). Multivariate analysis indicated that clinical stage status, platelet (PLT), and the minimum dose of gross tumor volume (BEDGTV_min_) were prognostic parameters for 1-year tumor local status.

**Table 2:** Univariate and multivariate analyses of clinical and dosimetric parameters.

Variable	Univariate analyses (logistic)		Multivariate analyses (logistic)		Multivariate analyses (stepwise)	
	*p*-value	*β*	*p*-value	*β*	*p*-value	*β*
Sex (man vs. woman)	0.299	−0.527				
Age (years)	0.615	−0.010				
Smoking status						
Current	Reference	Reference				
Former	0.990	−17.806				
Never	0.302	−0.506				
KPS (<80 vs. ≥80)	0.080	1.256				
BMI (kg/m^2^)	0.914	0.008				
Clinical stage (I~II vs. III~IV)	0.001	−2.132	0.214	−1.284	0.034	−1.543
Location (central vs. peripheral)	0.252	−0.784				
Histology						
Adenocarcinoma	Reference	Reference	Reference	Reference		
Squamous cell carcinoma	0.405	−0.597	0.733	−0.425		
Unknown	0.020	−1.477	0.931	0.101		
Equivalent diameter (cm)	0.197	−0.177				
GTV (cm^2^)	0.184	−0.005				
PTV (cm^2^)	0.140	−0.004				
Chemotherapy (yes vs. no)	0.431	0.493				
Lymphocyte (10^9^/L)	0.056	0.698				
Neutrophil (10^9^/L)	0.176	−0.134				
PLT (10^9^/L)	0.006	−0.010	0.115	−0.009	0.038	−0.009
NLR	0.128	−0.135				
PLR	0.011	−0.008	0.435	−0.004		
Hb (g/L)	0.031	0.037	0.376	0.021		
Immobilization device (vacuum bag vs. thermoplastic mask)	0.144	0.693				
4DCT (yes vs. no)	−0.851	0.109				
BED_95_ (<84.00 vs. ≥ 84.00) (Gy)	0.001	1.723	0.659	0.530		
BED_max_ (<110.85 vs. ≥110.85) (Gy)	<0.0001	2.431	0.992	14.790		
BEDPTV_min_ (<80.43 vs. ≥80.43) (Gy)	0.004	1.484	0.396	1.386		
BEDPTV_max_ (<110.85 vs. ≥110.85) (Gy)	<0.0001	2.351	0.992	−14.290		
BEDPTV_mean_ (<101.73 vs. ≥101.73) (Gy)	<0.0001	2.128	0.727	0.616		
BEDGTV_min_ (<98.79 vs. ≥98.79) (Gy)	<0.0001	2.258	0.510	0.848	0.009	1.699
BEDGTV_max_ (<103.87 vs. ≥103.87) (Gy)	<0.0001	1.983	0.891	−0.266		
BEDGTV_mean_ (<97.06 vs. ≥97.06) (Gy)	<0.0001	1.864	0.433	−1.752		
BEDPTV_min_/PTV_max_	0.004	−11.277	0.062	−13.31	0.071	−7.661
BEDGTV_min_/GTV_max_	0.075	−12.134				
Tumor site (primary vs. secondary)	0.398	−0.399				

KPS, Karnofsky performance status; BMI, body mass index; PTV, planning target volume; GTV, gross tumor volume; PLT, platelet; Hb, hemoglobin; NLR, neutrophil-to-lymphocyte ratio; PLR, platelet-to-lymphocyte ratio; 4DCT, four-dimensional computed tomography; BED_95_, the prescription dose covers 95% of the target area expressed as BED; BED_max_, the maximum dose in the whole plan; BEDPTV_min_, the minimum dose of PTV; BEDPTV_mean_, mean dose of PTV; BEDPTV_max_, the maximum dose of PTV; BEDPTV_min_/PTV_max_, dose inhomogeneity in PTV; BEDGTV_min_, the minimum dose of GTV; BEDGTV_mean_, mean dose of GTV; BEDGTV_max_, the maximum dose of GTV; BEDGTV_min_/GTV_max_, dose inhomogeneity in GTV.

### Radiomics Feature Selection

**Figure 3:**
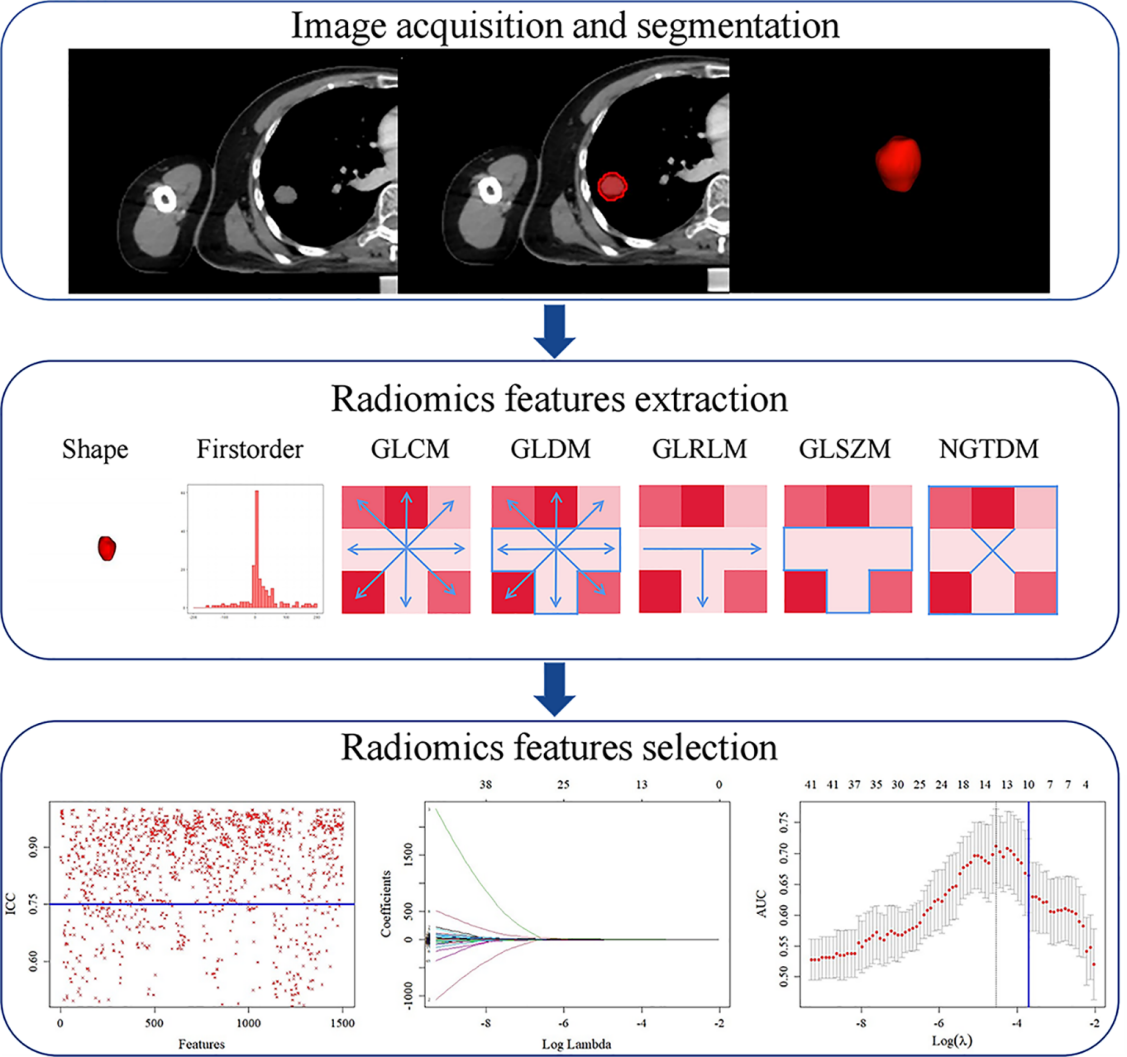
Radiomics feature extraction and selection process. First, region of interest (ROI) segmentation was performed on CT image. Next, radiomics features were extracted from ROI. Finally, radiomics features dimension was reduced by intraclass correlation coefficient (ICC), least absolute shrinkage and selection operator (LASSO). GLCM, gray-level co-occurrence matrix; GLDM, gray-level dependence matrix; GLRLM, gray-level run length matrix; GLSZM, gray-level size zone matrix; NGTDM, neighborhood gray-tone difference matrix.

**Figure 4:**
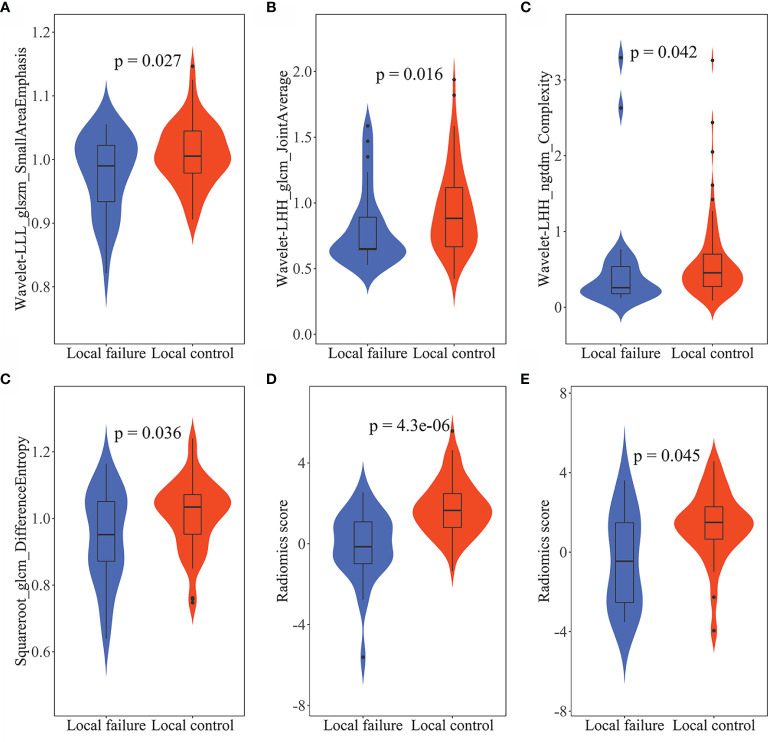
The violin plots of radiomics features and radiomics score. The distribution of **(A)** wavelet-LLL_glszm_SmallAreaEmphasis, **(B)** wavelet-LHH_glcm_JointAverage, **(C)** wavelet-LHH_ngtdm_Complexity, and **(D)** squareroot_glcm_DifferenceEntropy in the training group. The distribution of radiomics score in the training group **(E)** and in the validation group **(F)**. The *p*-values were obtained by *t*-test or Wilcoxon rank sum test.

**Figure 5:**
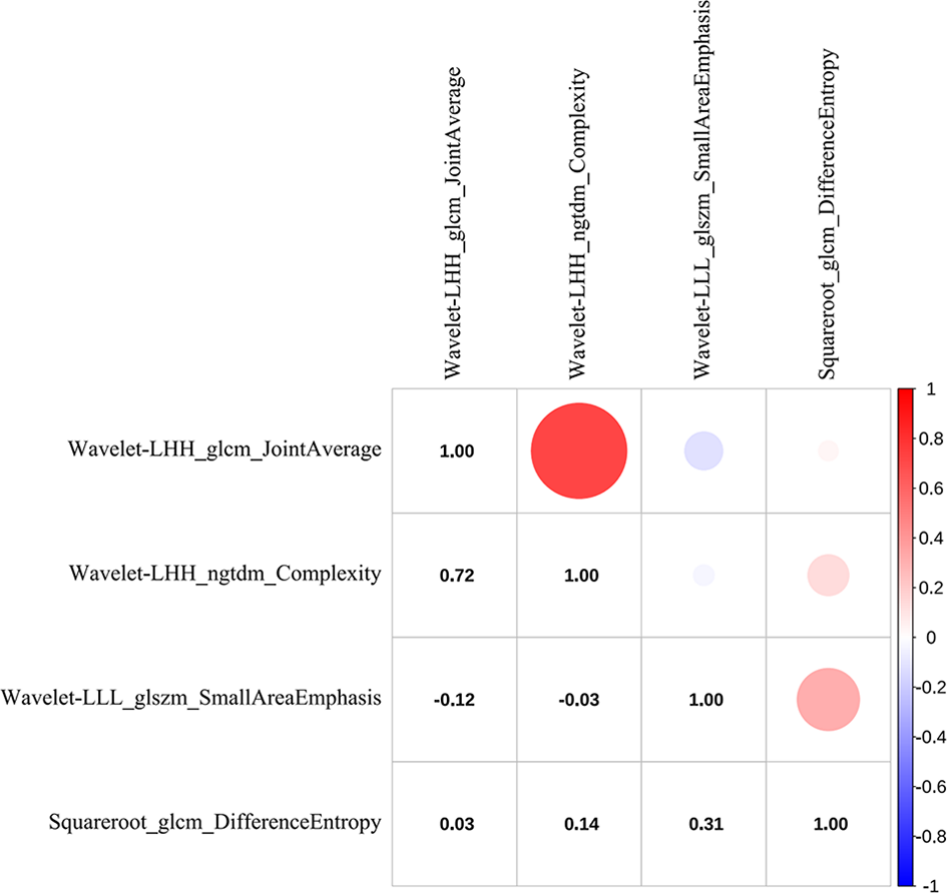
The correlation heat map of radiomics features. Red indicates positive correlation, and blue indicates negative correlation; the darker the color, the stronger the relationship.

Radiomics features were extracted and selected using the procedure shown in **Figure 3**. In total, 1,502 radiomics features were successfully extracted from each three-dimensional ROI, including 14 shape features, 288 first-order features, and 1,200 texture features. For intraobserver agreement, 1,090 features with ICC ≧0.75 between observers were included in further analyses. According to the univariate analysis, 46 radiomics features were collected, and then 10 potential radiomics features were calculated by the LASSO regression model with a penalty parameter *λ* = 0.025; we finally performed the stepwise regression analysis and obtained 4 important radiomics features, namely wavelet-LLL_glszm_SmallAreaEmphasis, wavelet-LHH_glcm_JointAverage, wavelet-LHH_ngtdm_Complexity, and squareroot_glcm_DifferenceEntropy. In the training group, the visible distributions of these radiomics features in local control group and local failure group and the correlation analysis of radiomics features are shown in **Figures 4**, **5**. It indicated that the larger the value of each radiomics features, the greater the possibility of 1-year tumor local control and they were statistically supported. There was no significant correlation between the radiomics features with correlation coefficient <0.75.

### Classifier Selection

The performance of model using difference classifiers are presented in **Table 3**. Our result reports that DT approach for classification were no more valuable than random classification with the AUC of the model below 0.5. We input these features into SVM classifier, which received the poor performance and the low specificity. Compared with the above methods, the LR was the optimum classifier with the best performance and accuracy.

**Table 3:** The performance of radiomics model using LR, DT, and SVM, clinical model and combined model.

Group	Methods	AUC (95% CI)	*p*-value	Accuracy (%)	Sensitivity (%)	Specificity (%)
Training	LR	0.811 (0.713–0.910)	0.000	67.4	57.1	92.3
	DT	0.832 (0.744–0.919)	1.000	90.9	79.4	80.8
	SVM	0.796 (0.691–0.901)	0.000	82.0	96.8	46.2
	Clinical	0.845 (0.757–0.934)	0.000	82.0	84.1	76.9
	Combined	0.911 (0.845–0.977)	0.000	85.4	87.3	80.8
Validation	LR	0.702 (0.507–0.898)	0.023	92.9	50.0	81.3
	DT	0.629 (0.429–0.830)	0.909	62.5	64.3	58.3
	SVM	0.714 (0.524–0.904)	0.018	75.0	85.7	50.0
	Clinical	0.786 (0.638–0.933)	0.002	77.5	78.6	75.0
	Combined	0.818 (0.659–0.978)	0.001	82.5	85.7	75.0

LR, logistic regression; DT, decision tree; SVM, support vector machine; AUC, area under the receiver operating characteristic curve (ROC); CI, confidence interval.

### Prediction Model Construction

On the basis of 4 radiomics factors, a radiomics model was created by the following formula:


$$\begin{array}{c} \text{radiomics score}=-27.645+14.393\\ \times \text{wavelet-LLL\_glszm\_SmallAreaEmphasis}\\ +8.075\times \text{wavelet-LHH\_glcm\_JointAverage}\\ -3.386\times \text{wavelet-LHH\_ngtdm\_Complexity}\\ +9.196\times \text{squareroot\_glcm\_DifferenceEntropy} \end{array}$$

The formula and these coefficients were calculated from the LR. To illustrate the validity of the radiomics score at the nomogram, the visible distributions of radiomics score for the 1-year tumor local control and local failure groups in the training group and validation group are shown in **Figure 4**. With the quantitative value of score increased, the tumor can be more possibly locally controlled in a year. The parameters of clinical stage status, PLT, and BEDGTV_min_ were employed to build the clinical model. Furthermore, the parameters plus radiomics score were brought into building the combined model.

### Prediction Model Performance Comparison

**Figure 6:**
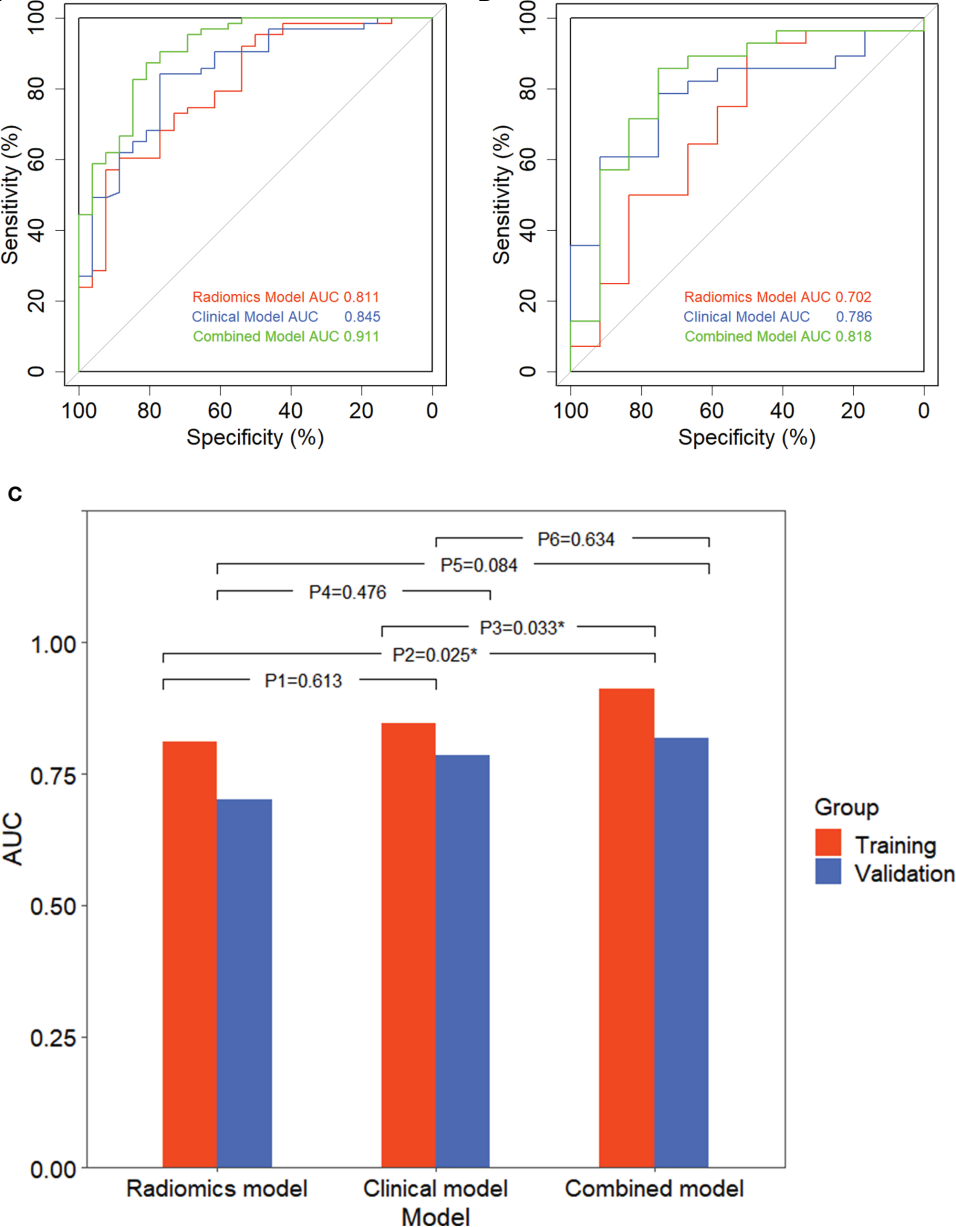
Receiver operating characteristic (ROC) curve of three models and comparison of ROC curves. ROC curve of three models in the training group **(A)** and validation group **(B)**. **(C)** Comparison of ROC curves with DeLong test in the training group and validation group.P1, P2, and P3 are in the training group; P4, P5, and P6 are in the validation group; P1 and P4: radiomics model vs. clinical model; P2 and P4: radiomics model vs. combined model; P3 and P6: clinical model vs. combined model. ^*^ *p* < 0.05, expressive significance.

**Figure 7:**
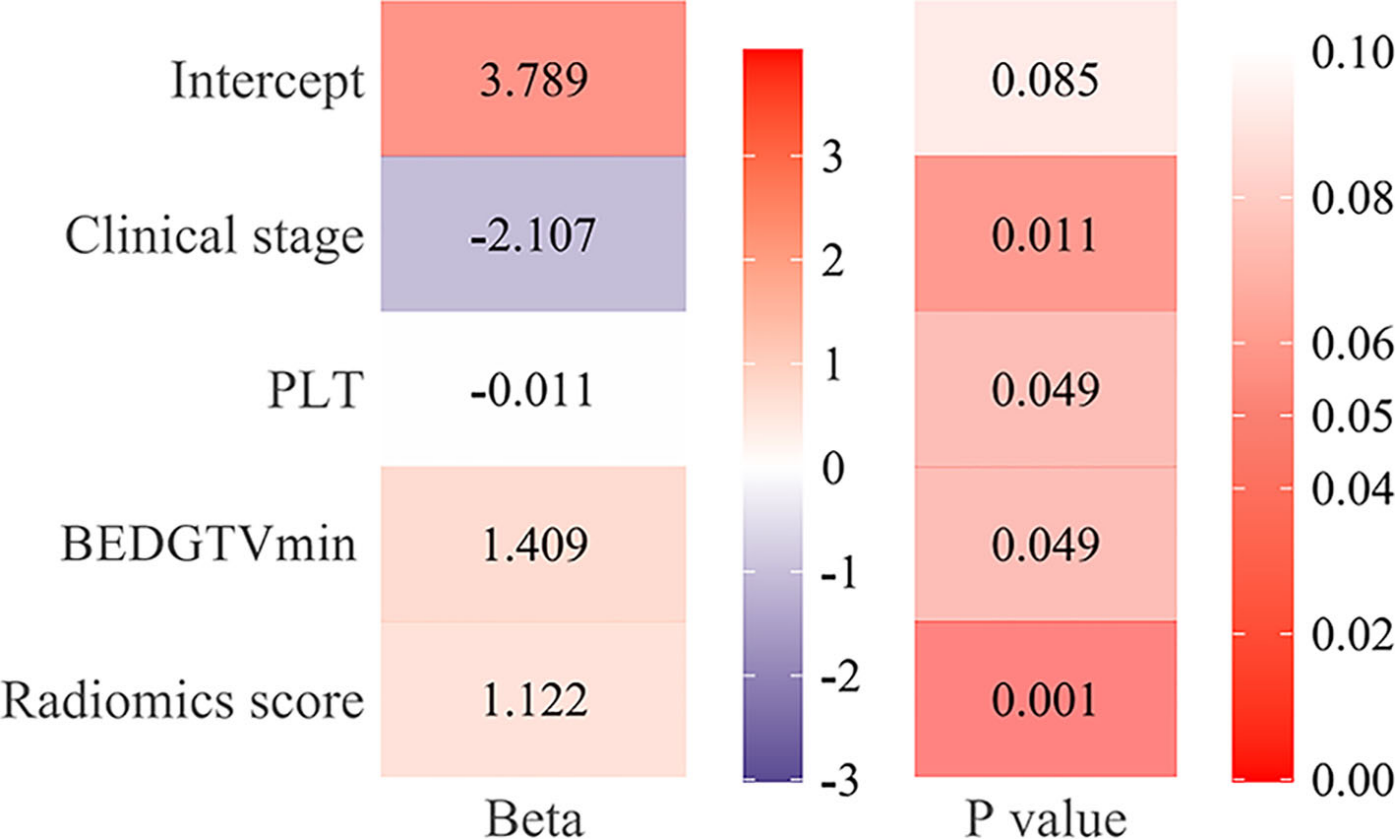
The function of each feature in the combined model. The beta value and the *p*-value of radiomics score, clinical stage [III~IV], platelet (PLT), and BEDGTV_min_ [≥98.79] in the combined model.

**Figure 6** showed that the AUC with its 95% confidence interval (CI) of the radiomics model, clinical model, and combined model were 0.811 (95% CI: 0.713–0.910), 0.845 (95% CI: 0.757–0.934), and 0.911 (95% CI: 0.845–0.977) in the training group and 0.702 (95% CI: 0.507–0.898), 0.786 (95% CI: 0.638–0.933), and 0.818 (95% CI: 0.659–0.978) in the validation group, respectively. The accuracy values of the radiomics model, clinical model, and combined model were 67.4%, 82.0% and 85.4% in the training group and 92.9%, 77.5%, and 82.5% in the validation group, respectively. The combined model indicated a significant better performance than the radiomics model (*p* = 0.025) and the clinical model (*p* = 0.033) in the training group, while the radiomics model and clinical model displayed a similar performance (*p* = 0.613). We can also see the trend that the effect of the combined model is better than that of the single model in the validation group. Therefore, the optimal prediction model was based on a multivariable LR and conjoined the radiomics signature with clinical and dosimetric parameters. Moreover, the contribution of each selected feature in the combined model iss displayed in **Figure 7**.

### Nomogram Establishment

**Figure 8:**
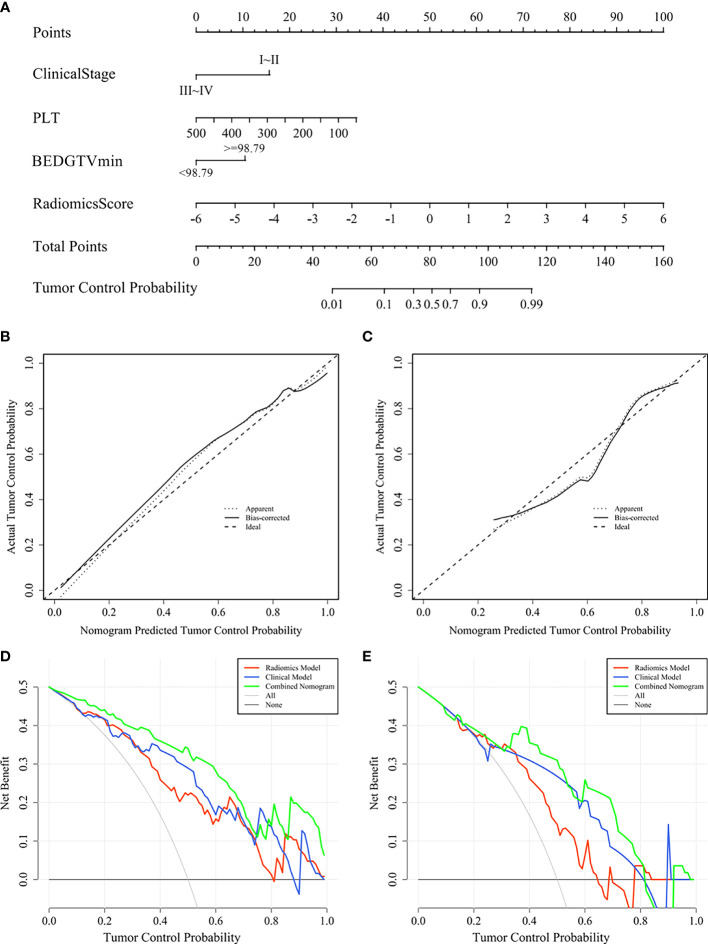
Combined nomogram and evaluation of the nomogram in the training group and validation group. **(A)** A combined nomogram for prediction 1-year local control probability in lung cancer patients after SBRT. The calibration curve of the combined nomogram in the training group **(B)** and validation group **(C)**. The decision curve of each model in the training group **(D)** and validation group **(E)**.

A visualization-combined nomogram was constructed from integrating the radiomics score, clinical stage, PLT, and BEDGTV_min_, as shown in **Figure 8A**. No significance was found in the Hosmer-Lemeshow test for the separated training sets (*p* = 0.898) and validation group (*p* = 0.891), indicating the proposed nomogram with good calibration was acceptable. The actual tumor control probability is that the patient population was divided into a few bins of increasing percentage of local control. The calibration curve of the combined nomogram confirmed that the probability of predicting 1-year tumor local control was consistent with the actual observation both in the training group (**Figure 8B**) and validation group (**Figure 8C**). The decision curve revealed the radiomics model, the clinical model, and the combined nomogram were beneficial for predicting 1-year tumor local control probability. The area under the curve of the combined nomogram was larger than that of the other two models, indicating that the combined nomogram had the highest clinical feasibility and utility (**Figures 8D, E**).

## Discussion

In this study, a quantitative relationship between radiomics score, clinical and dosimetric features, and tumor local status was found. Moreover, we first established a new prediction model to correlate 1-year local control with radiomics score and clinical and dosimetric parameters for primary and secondary lung cancer patients undergoing SBRT. We also constructed and validated a combined nomogram with great discrimination to conveniently identify the tumor local status.

Improving the accuracy of local control prediction is of positive significance for medical and personal decision-making in many aspects (2). It is beneficial to find patients who are at high risk of locoregional failure and explore the treatment strategy of patients. Moreover, increasing systemic treatment and/or radiation dose to eradicate lesions and strengthening the follow-up management of patients can reduce local recurrence and improve the survival and prognosis. In clinical practice, some studies have proposed to overcome immune resistance mechanisms for lung cancer by using immunotherapy combined with SBRT; therefore, patients have more personalized treatment options (35, 36). Our nomogram model may provide important evidence to design future clinical trials, such as predicting whether these people would benefit from combination treatment to balance this positive strategic risk. More importantly, compared with the long-term outcome of overall survival, local control status avoids long-term follow-up and can early adjust treatment strategies. Therefore, our study provides a more effective tool to promptly achieve personalized treatment.

The correlation between radiomics features and SBRT outcomes has shown promising results. Lafata et al. investigated the relationship between pre-SBRT CT radiomics features and cancer recurrence for nonsmall cell lung cancer (NSCLC) and concluded that radiomics features may provide more predictive information in the identification of tumor local failures (37). Mattonen et al. compared the prognostic value of physician and radiomics data for local response of NSCLC treated with SBRT. Their findings similarly indicated that a radiomics score consisting of five appearance features can early and correctly predict local recurrence in a noninvasive way (18). Our results are consistent with these published studies (18, 20, 21, 37), as we found radiomics features were independent prognostic factors and radiomics score was significantly associated with tumor local status for primary and secondary lung cancer undergoing SBRT. These results may explain that radiomics has the ability to quantify tumor spatial and temporal heterogeneity by mathematically analyzing the spatial distribution and relationships of gray levels in CT images.

Many studies have focused on the effects of clinical and dosimetric parameters on local control (9, 10); however, no consensus has been reached so far and further investigation is needed. Ohri et al. developed a local TCP model with BED and tumor diameter for NSCLC patients after SBRT (14), while Ye et al. established a nomogram model with tumor size and SUVmax to predict 2-year locoregional recurrence and 2-year progression-free survival (7). However, their analysis lacked additional SBRT datasets for reliability verification and the SUVmax based on PET-CT is not easy to obtain. Our study avoided these limitations and showed that clinical stage, PLT value, and BEDGTV_min_ were significantly correlated with the local control status. This finding is also in accordance with other studies (38–40). These results suggest that earlier clinical stage, lower PLT value and higher BEDGTV_min_ contribute to tumor local control and should be considered when designing SBRT regimens.

However, these previous studies mainly focused on radiomics features (18, 20, 21, 37) or clinical and dosimetric parameters (7, 14, 15, 41). In our work, we combined radiomics features and the available clinical and dosimetric parameters to improve the prediction performance and accuracy for local control in lung SBRT. The combined model indicated outstanding performance in the training group and had good stability in the validation group. It failed to achieve statistical significance due to the small sample size, subtle differences in the data set or the mixed effect of other parameters. Similar results were obtained by Avanzo et al. who have demonstrated that combining BED features and image features in radiomics and deep learning improves the tumor response prediction of machine learning models for lung SBRT (42). This trend is in agreement with past studies, showing it is highly valuable to predict tumor local control in lung SBRT using multivariate factors (43). Meanwhile, the combined nomogram-integrated multiple features increases the value of personalized estimation and has great clinical application potential (19). More importantly, the variables involved in nomogram are derived from clinically available data without the need for additional expense, which will increase the clinical applicability.

It is worth noting that the incidence of local control in our study is lower than that reported previously. It might be due to the inconsistent design of treatment plans and the selection bias between different studies. According to our nomogram, the radiation dose should be increased for patients with a high risk of local recurrence. However, considering the patient’s condition and nearby organs at risk, the BED_95_ used in our cohort, at a median of 95.2 Gy, was lower than the standard dose of 100 Gy (2). In addition, some metastatic lung cancer patients were treated with significantly lower radiation doses, such as 20~35 Gy in 4~7 fractions. This was probably the reason for the high local recurrence rate in our study. In our univariate analysis, BED_95_ was also a significant parameter. Due to the multicollinearity between dosimetric parameters, BED_95_ was removed from the final model by the stepwise regression methods. However, the last dosimetric parameters entering the model, namely BEDGTV_min_, covered some prediction information of the BED_95_. Also with more data, a continued work on verifying these results is imperative.

Finally, efforts have been made to reduce the risk of radiomics feature biases and improve the quality of the prediction models. A wide range of candidate radiomics features were extracted in our study, which provided the foundation for algorithms to select relevant radiomics features and obtained the valuable information to reflect the local control status of lung cancer lesions (24). In order to reduce the deviation of the interobservers and examine the feature stability, we calculated the ICC of radiomics features (44). In addition, we optimized feature selection by using univariate analysis, LASSO, and stepwise regression, thus ensuring the independence and robustness of each feature entering the final prediction model (26). Three popular classifiers were utilized to evaluate the performance of the radiomics model, and finally the classifier with the best accuracy and the highest prognostic performance was used to establish the prediction model (29, 30).

There are some limitations that should be considered in the study (1): Our study was a retrospective, single-center-based study and limited number of patients were involved in the study, and results from a prospective multicenter study with a greater number of population are needed (2). Local tumor failure was not pathologically confirmed by biopsy in the study, which added more uncertainty to our conclusions. However, data from the literature show that histological confirmation was not mandatory in NSCLC patients treated with SBRT (45). (3) Our study analyzed the tumor local control status from a mixture of primary and secondary lung tumor patients. However, it was found in the previous study that TCP models were not different between primary NSCLC and secondary NSCLC, because histological heterogeneity does not influence radiosensitivity of tumor in the SBRT (15). (4) Due to the limited sample size, our endpoint mainly focused on 1-year local control and further work is required to conduct a longer follow-up time and verify the practicability of the prediction model.

## Conclusions

We found that there was a significant quantitative correlation between radiomics score and local control for patients undergoing SBRT, and we consider that it might be a promising and potential biomarker. According to the LR method, we developed a novel model using radiomics score plus clinical and dosimetric parameters to improve the prediction of local control. The nomogram we established have a potential to be a noninvasive, low-cost approach and could facilitate individualized treatment and follow-up, surveillance, and evaluation strategies for patients undergoing SBRT.

## Data Availability Statement

The raw data supporting the conclusions of this article will be made available by the authors, without undue reservation.

## Author Contributions

The author’s responsibilities are as follows: B-TH, C-ZC, and L-ML conceived and designed the study. C-HS, L-ML, YW, R-HW, KH, and Z-HQ contributed to data collection. L-ML, G-BP, C-BZ, and Y-XW analyzed data and interpreted THE data. L-ML and B-TH wrote the paper. B-TH had primary responsibility for final content. All authors contributed to the manuscript review and approved the final version.

## Funding

This work was sponsored by THE National Natural Science Foundation of China (81602667), Medical Scientific Research Foundation of Guangdong Province (A2015534, B2016048), and the Creative and Facilitating Program of Shantou University.

## Conflict of Interest

The authors declare that the research was conducted in the absence of any commercial or financial relationships that could be construed as a potential conflict of interest.

## Publisher’s Note

All claims expressed in this article are solely those of the authors and do not necessarily represent those of their affiliated organizations, or those of the publisher, the editors and the reviewers. Any product that may be evaluated in this article, or claim that may be made by its manufacturer, is not guaranteed or endorsed by the publisher.

## Glossary

**Table:**

SBRT	stereotactic body radiation therapy
CT	Computed tomography
SUVmax	maximum standardized uptake value
4DCT	four-dimensional computed tomography
3DCT	three-dimensional computed tomography
CBCT	Cone beam computed tomography
GTV	gross tumor volume
ITV	internal target volume
PTV	planning target volume
BED	biologically effective dose
KPS	Karnofsky performance status
BMI	body mass index
PLT	platelet
Hb	hemoglobin
NLR	neutrophil-to-lymphocyte ratio
PLR	platelet-to-lymphocyte ratio
BED_95_	the prescription dose covers 95% of the target area expressed as BED
BED_max_	the maximum dose in the whole plan
BEDPTV_min_	the minimum dose of PTV
BEDPTV_mean_	mean dose of PTV
BEDPTV_max_	the maximum dose of PTV
BEDPTV_min_/PTV_max_	dose inhomogeneity in PTV
BEDGTV_min_	the minimum dose of GTV
BEDGTV_mean_	mean dose of GTV
BEDGTV_max_	the maximum dose of GTV
BEDGTV_min_/GTV_max_	dose inhomogeneity in GTV
ROI	region of interest
GLCM	gray-level co-occurrence matrix
GLDM	gray-level dependence matrix
GLRLM	gray-level run length matrix
GLSZM	gray-level size zone matrix
NGTDM	neighborhood gray-tone difference matrix
ICC	intraclass correlation coefficient
LASSO	least absolute shrinkage and selection operator
LR	logistic regression
DT	decision tree
SVM	support vector machine
ROC	receiver operating characteristic
AUC	area under the ROC curve
CI	confidence interval
NSCLC	nonsmall cell lung cancer
TCP	tumor control probability
LVI	lymphovascular invasion
PET-CT	positron emission tomography-CT
