# Supplementary material for: A Combined Model to Improve the Prediction of Local Control for Lung Cancer Patients Undergoing Stereotactic Body Radiotherapy Based on Radiomic Signature Plus Clinical and Dosimetric Parameters
Source: Front Oncol. 2022 Jan 31;11:819047. doi: 10.3389/fonc.2021.819047 (PMC8841423; doi:10.3389/fonc.2021.819047)
Supplement: Supplementary file 1 [file Table_1.docx]

**Caption for supplementary material**

**Supplementary tables**

**Table S1. Clinical and dosimetric characteristics of primary and secondary lung cancer patients.**

| Characteristics | Overall | Training | Validation | P value |
| --- | --- | --- | --- | --- |
|  | Counts (%)/  Mean(±SD) | Counts (%)/  Mean(±SD) | Counts (%)/  Mean(±SD) |  |
| Gender |  |  |  | 0.722 |
| Man | 93(72.1) | 65(73.0) | 28(70.0) |  |
| Woman | 36(27.9) | 24(27.0) | 12(30.0) |  |
| Age [years] | 62(±12) | 63(±12) | 60(±11) | 0.230 |
| Smoking status |  |  |  | 0.512 |
| Current | 67(51.9) | 49(55.0) | 18(45.0) |  |
| Former | 4(3.1) | 3(3.4) | 1(2.5) |  |
| Never | 58(45.0) | 37(41.6) | 21(52.5) |  |
| KPS |  |  |  | 0.056 |
| <80 | 9(7.0) | 9(10.1) | 0(0.0) |  |
| ≧80 | 120(93.0) | 80(89.9) | 40(100.0) |  |
| BMI [kg/m^2^] | 21.53(±2.86) | 21.54(±3.05) | 21.49(±2.43) | 0.873 |
| Clinical stage |  |  |  | 0.557 |
| I~II | 50(38.8) | 36(40.4) | 14(35.0) |  |
| III~IV | 79(61.2) | 53(59.6) | 26(65.0) |  |
| Location |  |  |  | 0.357 |
| Central | 22(17.1) | 17(19.1) | 5(12.5) |  |
| Peripheral | 107(82.9) | 72(80.9) | 35(87.5) |  |
| Histology |  |  |  | 0.678 |
| Adenocarcinoma | 42(32.6) | 27(30.3) | 15(37.5) |  |
| Squamous cell carcinoma | 34(26.4) | 25(28.1) | 9(22.5) |  |
| Unknown | 53(41.1) | 37(41.6) | 16(40.0) |  |
| Equivalent diameter[cm] | 3.11(±1.70) | 3.00(±1.66) | 3.37(±1.76) | 0.184 |
| GTV [cm^2^] | 32.23(±56.75) | 29.56(±56.46) | 38.18(±57.66) | 0.204 |
| PTV [cm^2^] | 80.89(±100.05) | 74.17(±95.08) | 95.85(±110.09) | 0.270 |
| Chemotherapy |  |  |  | 0.082 |
| Yes | 24(18.6) | 13(14.6) | 11(27.5) |  |
| No | 105(81.4) | 76(85.4) | 29(72.5) |  |
| Lymphocyte [10^9^/L] | 1.64(±0.73) | 1.68(±0.75) | 1.55(±0.67) | 0.500 |
| Neutrophil [10^9^/L] | 4.30(±2.33) | 4.28(±2.29) | 4.33(±2.45) | 0.913 |
| PLT [10^9^/L] | 220.50(±71.71) | 221.50(±72.41) | 218.30(±70.99) | 0.676 |
| NLR | 3.24(±2.97) | 3.27(±3.41) | 3.15(±1.62) | 0.310 |
| PLR | 163.73(±101.99) | 160.85(±105.46) | 170.12(±94.78) | 0.588 |
| Hb [g/L] | 127.10(±17.10) | 128.50(±14.03) | 124.10(±22.40) | 0.402 |
| Immobilization device |  |  |  | 0.321 |
| Vacuum bag | 53(41.1) | 34(38.2) | 19(47.5) |  |
| Thermoplastic mask | 76(58.9) | 55(61.8) | 21(52.5) |  |
| 4DCT |  |  |  |  |
| Yes | 49(38.0) | 32(36.0) | 17(42.5) | 0.479 |
| No | 80(62.0) | 57(64.0) | 23(57.5) |  |
| BED_95_ [Gy] |  |  |  | 0.886 |
| <84.00 | 44(34.1) | 30(33.7) | 14(35.0) |  |
| ≧84.00 | 85(65.9) | 59(66.3) | 26(65.0) |  |
| BED_max_ [Gy] |  |  |  | 0.611 |
| <110.85 | 57(44.2) | 38(42.7) | 19(47.5) |  |
| ≧110.85 | 72(55.8) | 51(57.3) | 21(52.5) |  |
| BEDPTV_min_ [Gy] |  |  |  | 0.482 |
| <80.43 | 65(50.4) | 43(48.3) | 22(55.0) |  |
| ≧80.43 | 64(49.6) | 46(51.7) | 18(45.0) |  |
| BEDPTV_max_ [Gy] |  |  |  | 0.698 |
| <110.85 | 58(45.0) | 39(43.8) | 19(47.5) |  |
| ≧110.85 | 71(55.0) | 50(56.2) | 21(52.5) |  |
| BEDPTV_mean_ [Gy] |  |  |  | 0.577 |
| <101.73 | 63(48.8) | 42(47.2) | 21(52.5) |  |
| ≧101.73 | 66(51.2) | 47(52.8) | 19(47.5) |  |
| BEDGTV_min_ [Gy] |  |  |  | 0.559 |
| <98.79 | 63(48.8) | 45(50.6) | 18(45.0) |  |
| ≧98.79 | 66(51.2) | 44(49.4) | 22(55.0) |  |
| BEDGTV_max_ [Gy] |  |  |  | 0.490 |
| <103.87 | 46(35.7) | 30(33.7) | 16(40.0) |  |
| ≧103.87 | 83(64.3) | 59(66.3) | 24(60.0) |  |
| BEDGTV_mean_ [Gy] |  |  |  | 0.350 |
| <97.06 | 41(31.8) | 26(29.2) | 15(37.5) |  |
| ≧97.06 | 88(68.2) | 63(70.8) | 25(62.5) |  |
| BEDPTV_min_/PTV_max_ | 0.80(±0.08) | 0.80(±0.09) | 0.80(±0.07) | 0.769 |
| BEDGTV_min_/GTV_max_ | 0.90(±0.09) | 0.90(±0.11) | 0.91(±0.04) | 0.882 |
| Tumor site |  |  |  | 0.640 |
| Primary | 80(62.0) | 54(60.7) | 26(65.0) |  |
| Secondary | 49(38.0) | 35(39.3) | 14(35.0) |  |
| 1-Year local control |  |  |  | 0.928 |
| Yes | 91(70.5) | 26(29.2) | 12(30.0) |  |
| No | 38(29.5) | 63(70.8) | 28(70.0) |  |

Chi square test, t test or Wilcoxon test were used to calculate the statistical differences between training group and validation group. Abbreviations: KPS, Karnofsky performance status; BMI, body mass index; PTV, planning target volume; GTV, gross tumor volume; PLT, platelet; Hb, hemoglobin; NLR, Neutrophil-to-Lymphocyte ratio; PLR, Platelet-to-Lymphocyte ratio; 4DCT, four-dimensional computed tomography; BED_95_, the prescription dose covers 95% of the target area expressed as BED; BED_max_, the maximum dose in the whole plan; BEDPTV_min_, the minimum dose of PTV; BEDPTV_mean_, mean dose of PTV; BEDPTV_max_, the maximum dose of PTV; BEDPTV_min_/PTV_max_, dose inhomogeneity in PTV; BEDGTV_min_, the minimum dose of GTV; BEDGTV_mean_, mean dose of GTV; BEDGTV_max_, the maximum dose of GTV; BEDGTV_min_/GTV_max_, dose inhomogeneity in GTV.
